# *N*′-(2-Meth­oxy­benzyl­idene)-4-nitro­benzohydrazide

**DOI:** 10.1107/S1600536811008014

**Published:** 2011-03-09

**Authors:** Hai-Yun Zhu

**Affiliations:** a Department of Chemistry and Chemical Engineering, Baoji University of Arts and Sciences, Baoji 721013, People’s Republic of China

## Abstract

The mol­ecule of the title compound, C_15_H_13_N_3_O_4_, adopts an *E* configuration with respect to the C=N bond. The dihedral angle between the two benzene rings is 6.0 (3)°. In the crystal, mol­ecules are linked through inter­molecular N—H⋯O hydrogen bonds to form chains along the *c* axis.

## Related literature

For background on hydrazone compounds, see: Rasras *et al.* (2010 ▶); Fan *et al.* (2010 ▶); Ajani *et al.* (2010 ▶); Avaji *et al.* (2009 ▶). For the crystal structures of typical hydrazone compounds, see: Khaledi *et al.* (2010 ▶); Han *et al.* (2010 ▶); Hussain *et al.* (2010 ▶); Ji & Lu (2010 ▶). For the hydrazone compound reported recently by the author, see: Zhu (2010 ▶). For the reference bond values, see: Allen *et al.* (1987 ▶).
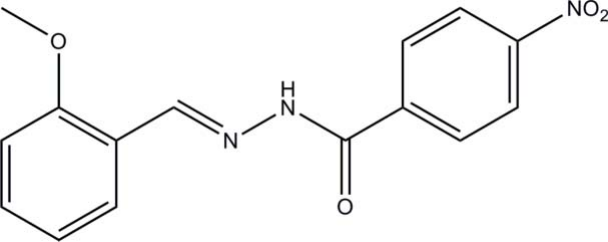


## Experimental

### 

#### Crystal data

C_15_H_13_N_3_O_4_
*M* *_r_* = 299.28
Monoclinic, 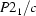

*a* = 10.737 (2) Å
*b* = 14.728 (2) Å
*c* = 9.132 (1) Å
β = 93.572 (2)°
*V* = 1441.3 (4) Å^3^
*Z* = 4
Mo *K*α radiation
μ = 0.10 mm^−1^
*T* = 298 K
0.23 × 0.21 × 0.20 mm

#### Data collection

Bruker SMART CCD area-detector diffractometer
Absorption correction: multi-scan (*SADABS*; Bruker, 2001 ▶) *T*_min_ = 0.977, *T*_max_ = 0.980
9434 measured reflections
3129 independent reflections
1426 reflections with *I* > 2σ(*I*)
*R*_int_ = 0.077

#### Refinement

*R*[*F* ^2^ > 2σ(*F* ^2^)] = 0.058
*wR*(*F* ^2^) = 0.154
*S* = 0.99
3129 reflections
203 parameters
1 restraint
H atoms treated by a mixture of independent and constrained refinement
Δρ_max_ = 0.16 e Å^−3^
Δρ_min_ = −0.20 e Å^−3^

### 

Data collection: *SMART* (Bruker, 2007 ▶); cell refinement: *SAINT* (Bruker, 2007 ▶); data reduction: *SAINT*; program(s) used to solve structure: *SHELXTL* (Sheldrick, 2008 ▶); program(s) used to refine structure: *SHELXTL*; molecular graphics: *SHELXTL*; software used to prepare material for publication: *SHELXTL*.

## Supplementary Material

Crystal structure: contains datablocks global, I. DOI: 10.1107/S1600536811008014/sj5112sup1.cif

Structure factors: contains datablocks I. DOI: 10.1107/S1600536811008014/sj5112Isup2.hkl

Additional supplementary materials:  crystallographic information; 3D view; checkCIF report

## Figures and Tables

**Table 1:** Hydrogen-bond geometry (Å, °)

*D*—H⋯*A*	*D*—H	H⋯*A*	*D*⋯*A*	*D*—H⋯*A*
N2—H2⋯O2^i^	0.90 (1)	2.04 (1)	2.913 (3)	165 (2)

Symmetry code: (i) 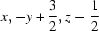.

**Fig. 1.**
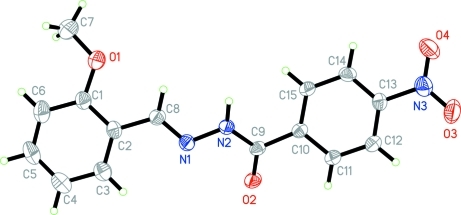
The molecular structure of the title compound with 30% probability displacement ellipsoids for non-hydrogen atoms.

**Fig. 2.**
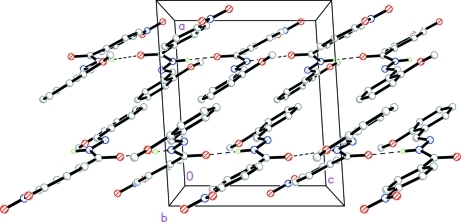
The molecular packing of the title compound. Hydrogen bonds are drawn as dashed lines.

## supplementary crystallographic information

### Comment

In recent years, considerable attention has been focused on the preparation and biological properties of hydrazone compounds (Rasras *et al.*, 2010; Fan *et al.*, 2010; Ajani *et al.*, 2010; Avaji *et al.*, 2009). The crystal structures of a number of hydrazone compounds have been reported (Khaledi *et al.*, 2010; Han *et al.*, 2010; Hussain *et al.*, 2010; Ji & Lu, 2010). As a continuation of the work on the structures of hydrazone compounds (Zhu, 2010), the author reports in this paper the title new hydrazone compound, Fig. 1.

The molecule of the compound adopts an *E* configuration with respect to the C═N bond. The dihedral angle between the C1—C6 and C10—C15 benzene rings is 6.0 (3)°. All the bond lengths are within normal values (Allen *et al.*, 1987), and are comparable with those in the similar hydrazone compounds as cited above. In the crystal structure, molecules are linked through intermolecular N—H···O hydrogen bonds (Table 1) to form chains along the *c* axis (Fig. 2).

### Experimental

2-Methoxybenzaldehyde (0.136 g, 1 mmol) and 4-nitrobenzohydrazide (0.181 g, 1 mmol) were dissolved in 30 ml absolute methanol. The mixture was stirred at reflux for 10 min, and cooled to room temperature. The clear yellow solution was left to slow evaporation in air for 3 d, yielding yellow needle crystals of the compound.

### Refinement

H2 attached to N2 was located from a difference Fourier map and refined isotropically, with the N—H distance restrained to 0.90 (1) Å. The remaining H atoms were positioned geometrically and refined using the riding-model approximation, with C—H = 0.93 or 0.96 Å, and *U*_iso_(H) = 1.2*U*_eq_(C) or *U*_iso_(H) = 1.5*U*_eq_(C7).

### Crystal data

**Table:**

C_15_H_13_N_3_O_4_	*F*(000) = 624
*M**_r_* = 299.28	*D*_x_ = 1.379 Mg m^−^^3^
Monoclinic, *P*2_1_/*c*	Mo *K*α radiation, λ = 0.71073 Å
*a* = 10.737 (2) Å	Cell parameters from 864 reflections
*b* = 14.728 (2) Å	θ = 2.3–24.5°
*c* = 9.132 (1) Å	µ = 0.10 mm^−^^1^
β = 93.572 (2)°	*T* = 298 K
*V* = 1441.3 (4) Å^3^	Cut from needle, yellow
*Z* = 4	0.23 × 0.21 × 0.20 mm

### Data collection

**Table:**

Bruker SMART CCD area-detector diffractometer	3129 independent reflections
Radiation source: fine-focus sealed tube	1426 reflections with *I* > 2σ(*I*)
graphite	*R*_int_ = 0.077
ω scans	θ_max_ = 27.0°, θ_min_ = 2.4°
Absorption correction: multi-scan (*SADABS*; Bruker, 2001)	*h* = −13→13
*T*_min_ = 0.977, *T*_max_ = 0.980	*k* = −15→18
9434 measured reflections	*l* = −11→11

### Refinement

**Table:**

Refinement on *F*^2^	Primary atom site location: structure-invariant direct methods
Least-squares matrix: full	Secondary atom site location: difference Fourier map
*R*[*F*^2^ > 2σ(*F*^2^)] = 0.058	Hydrogen site location: inferred from neighbouring sites
*wR*(*F*^2^) = 0.154	H atoms treated by a mixture of independent and constrained refinement
*S* = 0.99	*w* = 1/[σ^2^(*F*_o_^2^) + (0.0427*P*)^2^] where *P* = (*F*_o_^2^ + 2*F*_c_^2^)/3
3129 reflections	(Δ/σ)_max_ < 0.001
203 parameters	Δρ_max_ = 0.16 e Å^−^^3^
1 restraint	Δρ_min_ = −0.20 e Å^−^^3^

### Special details

**Table:**

Geometry. All e.s.d.'s (except the e.s.d. in the dihedral angle between two l.s. planes) are estimated using the full covariance matrix. The cell e.s.d.'s are taken into account individually in the estimation of e.s.d.'s in distances, angles and torsion angles; correlations between e.s.d.'s in cell parameters are only used when they are defined by crystal symmetry. An approximate (isotropic) treatment of cell e.s.d.'s is used for estimating e.s.d.'s involving l.s. planes.
Refinement. Refinement of *F*^2^ against ALL reflections. The weighted *R*-factor *wR* and goodness of fit *S* are based on *F*^2^, conventional *R*-factors *R* are based on *F*, with *F* set to zero for negative *F*^2^. The threshold expression of *F*^2^ > σ(*F*^2^) is used only for calculating *R*-factors(gt) *etc*. and is not relevant to the choice of reflections for refinement. *R*-factors based on *F*^2^ are statistically about twice as large as those based on *F*, and *R*- factors based on ALL data will be even larger.

### Fractional atomic coordinates and isotropic or equivalent isotropic displacement parameters (Å^2^)

**Table:**

	*x*	*y*	*z*	*U*_iso_*/*U*_eq_	
N1	0.30611 (19)	0.83021 (15)	1.0924 (2)	0.0485 (6)	
N2	0.2592 (2)	0.75864 (15)	1.0066 (2)	0.0494 (6)	
N3	0.0510 (2)	0.38411 (19)	0.7154 (3)	0.0661 (7)	
O1	0.27693 (19)	1.07382 (12)	0.9099 (2)	0.0761 (7)	
O2	0.22796 (17)	0.67342 (11)	1.2070 (2)	0.0597 (6)	
O3	0.0703 (2)	0.30882 (15)	0.7635 (3)	0.1087 (10)	
O4	−0.0029 (2)	0.39878 (16)	0.5963 (3)	0.0971 (8)	
C1	0.3456 (2)	1.07183 (19)	1.0414 (3)	0.0539 (7)	
C2	0.3634 (2)	0.98652 (18)	1.1036 (3)	0.0469 (7)	
C3	0.4307 (2)	0.9800 (2)	1.2379 (3)	0.0612 (8)	
H3	0.4428	0.9234	1.2817	0.073*	
C4	0.4799 (3)	1.0566 (2)	1.3077 (4)	0.0776 (10)	
H4	0.5248	1.0516	1.3977	0.093*	
C5	0.4618 (3)	1.1399 (2)	1.2431 (4)	0.0752 (10)	
H5	0.4958	1.1912	1.2896	0.090*	
C6	0.3947 (3)	1.1492 (2)	1.1112 (4)	0.0695 (9)	
H6	0.3821	1.2062	1.0691	0.083*	
C7	0.2470 (4)	1.1582 (2)	0.8462 (4)	0.1186 (16)	
H7A	0.2078	1.1957	0.9160	0.178*	
H7B	0.1908	1.1496	0.7614	0.178*	
H7C	0.3218	1.1872	0.8176	0.178*	
C8	0.3139 (2)	0.90618 (17)	1.0267 (3)	0.0476 (7)	
H8	0.2874	0.9105	0.9280	0.057*	
C9	0.2221 (2)	0.68303 (17)	1.0733 (3)	0.0439 (6)	
C10	0.1746 (2)	0.60759 (16)	0.9749 (3)	0.0408 (6)	
C11	0.1895 (2)	0.51927 (17)	1.0271 (3)	0.0498 (7)	
H11	0.2268	0.5096	1.1204	0.060*	
C12	0.1499 (2)	0.44613 (18)	0.9432 (3)	0.0524 (7)	
H12	0.1615	0.3871	0.9775	0.063*	
C13	0.0927 (2)	0.46272 (17)	0.8069 (3)	0.0467 (7)	
C14	0.0734 (2)	0.54826 (19)	0.7530 (3)	0.0507 (7)	
H14	0.0332	0.5573	0.6610	0.061*	
C15	0.1148 (2)	0.62158 (18)	0.8380 (3)	0.0492 (7)	
H15	0.1023	0.6804	0.8029	0.059*	
H2	0.251 (2)	0.7686 (17)	0.9092 (12)	0.059*	

### Atomic displacement parameters (Å^2^)

**Table:**

	*U*^11^	*U*^22^	*U*^33^	*U*^12^	*U*^13^	*U*^23^
N1	0.0614 (13)	0.0449 (13)	0.0394 (13)	−0.0007 (11)	0.0049 (11)	−0.0043 (12)
N2	0.0729 (15)	0.0427 (14)	0.0324 (12)	−0.0021 (11)	0.0011 (12)	−0.0001 (12)
N3	0.0642 (16)	0.0621 (18)	0.071 (2)	−0.0084 (13)	0.0000 (14)	−0.0067 (16)
O1	0.1061 (16)	0.0448 (13)	0.0748 (15)	0.0013 (11)	−0.0154 (13)	−0.0005 (11)
O2	0.0974 (15)	0.0493 (11)	0.0326 (11)	0.0034 (10)	0.0066 (10)	0.0039 (9)
O3	0.120 (2)	0.0480 (14)	0.151 (3)	0.0031 (13)	−0.0509 (18)	−0.0146 (16)
O4	0.140 (2)	0.0878 (18)	0.0610 (16)	−0.0348 (15)	−0.0160 (15)	−0.0079 (14)
C1	0.0560 (17)	0.0469 (18)	0.0589 (19)	−0.0005 (13)	0.0028 (15)	−0.0070 (15)
C2	0.0471 (15)	0.0471 (17)	0.0473 (17)	−0.0030 (12)	0.0094 (13)	−0.0101 (14)
C3	0.0607 (18)	0.062 (2)	0.061 (2)	−0.0022 (15)	0.0040 (16)	−0.0078 (16)
C4	0.069 (2)	0.088 (3)	0.074 (2)	−0.0099 (19)	−0.0092 (18)	−0.015 (2)
C5	0.0661 (19)	0.069 (2)	0.089 (3)	−0.0102 (17)	−0.0021 (19)	−0.031 (2)
C6	0.0653 (19)	0.0504 (19)	0.093 (3)	−0.0031 (15)	0.0068 (19)	−0.0135 (18)
C7	0.178 (4)	0.057 (2)	0.114 (3)	0.000 (2)	−0.052 (3)	0.016 (2)
C8	0.0585 (16)	0.0463 (16)	0.0385 (16)	0.0028 (13)	0.0077 (13)	−0.0005 (14)
C9	0.0539 (15)	0.0426 (16)	0.0358 (16)	0.0080 (12)	0.0070 (12)	0.0043 (14)
C10	0.0444 (14)	0.0433 (16)	0.0357 (15)	0.0039 (12)	0.0101 (12)	0.0044 (13)
C11	0.0588 (16)	0.0453 (17)	0.0449 (16)	0.0023 (13)	−0.0001 (13)	0.0058 (14)
C12	0.0585 (17)	0.0434 (16)	0.0551 (18)	0.0034 (13)	0.0015 (15)	0.0081 (15)
C13	0.0481 (15)	0.0426 (16)	0.0502 (17)	−0.0058 (12)	0.0089 (13)	−0.0046 (14)
C14	0.0591 (17)	0.0574 (18)	0.0356 (15)	−0.0037 (14)	0.0029 (13)	0.0050 (15)
C15	0.0632 (17)	0.0441 (16)	0.0402 (17)	0.0008 (13)	0.0017 (14)	0.0065 (14)

### Geometric parameters (Å, °)

**Table:**

N1—C8	1.275 (3)	C5—C6	1.371 (4)
N1—N2	1.389 (3)	C5—H5	0.9300
N2—C9	1.342 (3)	C6—H6	0.9300
N2—H2	0.900 (10)	C7—H7A	0.9600
N3—O3	1.206 (3)	C7—H7B	0.9600
N3—O4	1.219 (3)	C7—H7C	0.9600
N3—C13	1.480 (3)	C8—H8	0.9300
O1—C1	1.370 (3)	C9—C10	1.499 (3)
O1—C7	1.402 (3)	C10—C15	1.385 (3)
O2—C9	1.227 (3)	C10—C11	1.391 (3)
C1—C2	1.387 (4)	C11—C12	1.374 (3)
C1—C6	1.393 (4)	C11—H11	0.9300
C2—C3	1.387 (4)	C12—C13	1.375 (4)
C2—C8	1.460 (3)	C12—H12	0.9300
C3—C4	1.384 (4)	C13—C14	1.364 (3)
C3—H3	0.9300	C14—C15	1.387 (3)
C4—C5	1.369 (4)	C14—H14	0.9300
C4—H4	0.9300	C15—H15	0.9300
			
C8—N1—N2	115.6 (2)	H7A—C7—H7B	109.5
C9—N2—N1	118.7 (2)	O1—C7—H7C	109.5
C9—N2—H2	124.7 (16)	H7A—C7—H7C	109.5
N1—N2—H2	116.4 (17)	H7B—C7—H7C	109.5
O3—N3—O4	123.3 (3)	N1—C8—C2	121.1 (2)
O3—N3—C13	118.3 (3)	N1—C8—H8	119.4
O4—N3—C13	118.3 (3)	C2—C8—H8	119.4
C1—O1—C7	118.7 (2)	O2—C9—N2	123.3 (2)
O1—C1—C2	115.5 (2)	O2—C9—C10	120.4 (2)
O1—C1—C6	123.4 (3)	N2—C9—C10	116.3 (2)
C2—C1—C6	121.0 (3)	C15—C10—C11	119.0 (2)
C1—C2—C3	118.4 (3)	C15—C10—C9	123.5 (2)
C1—C2—C8	120.0 (3)	C11—C10—C9	117.4 (2)
C3—C2—C8	121.6 (3)	C12—C11—C10	121.2 (3)
C4—C3—C2	120.9 (3)	C12—C11—H11	119.4
C4—C3—H3	119.6	C10—C11—H11	119.4
C2—C3—H3	119.6	C11—C12—C13	118.1 (3)
C5—C4—C3	119.5 (3)	C11—C12—H12	121.0
C5—C4—H4	120.3	C13—C12—H12	121.0
C3—C4—H4	120.3	C14—C13—C12	122.7 (2)
C4—C5—C6	121.4 (3)	C14—C13—N3	119.0 (3)
C4—C5—H5	119.3	C12—C13—N3	118.3 (3)
C6—C5—H5	119.3	C13—C14—C15	118.8 (2)
C5—C6—C1	118.9 (3)	C13—C14—H14	120.6
C5—C6—H6	120.6	C15—C14—H14	120.6
C1—C6—H6	120.6	C10—C15—C14	120.3 (2)
O1—C7—H7A	109.5	C10—C15—H15	119.9
O1—C7—H7B	109.5	C14—C15—H15	119.9

### Hydrogen-bond geometry (Å, °)

**Table:**

*D*—H···*A*	*D*—H	H···*A*	*D*···*A*	*D*—H···*A*
N2—H2···O2^i^	0.90 (1)	2.04 (1)	2.913 (3)	165 (2)

Symmetry codes: (i) *x*, −*y*+3/2, *z*−1/2.
